# Analysis of the Responsiveness of Latanoprost, Travoprost, Bimatoprost, and Tafluprost in the Treatment of OAG/OHT Patients

**DOI:** 10.1155/2021/5586719

**Published:** 2021-05-25

**Authors:** Ziyan Cai, Mengdan Cao, Ke Liu, Xuanchu Duan

**Affiliations:** ^1^Department of Ophthalmology, The Second Xiangya Hospital of Central South University, Changsha, Hunan, China; ^2^Department of Ophthalmology, Changsha Aier Eye Hospital, Changsha, Hunan, China

## Abstract

**Aim:**

Within the clinical setting, some patients have been identified as lacking in response to PGAs. This meta-analysis study aimed to evaluate the responsiveness of latanoprost, travoprost, bimatoprost, and tafluprost in OAG/OHT patients, latanoprost nonresponders (LNRs), and the IOP-reducing efficacy and safety.

**Methods:**

A literature search was conducted on PubMed, Embase, and the Cochrane Controlled Trials Register. The primary clinical endpoint was the number of responders at the end of the study. The secondary clinical endpoint was the IOP reduction at the endpoint from baseline. Safety evaluation included five common adverse events: conjunctival hyperemia, hypertrichosis, ocular burning, ocular itching, and foreign-body sensation.

**Results:**

Eleven articles containing ten RCTs were included in this meta-analysis study. The results highlighted that, in the OAG/OHT population, there was no statistically significant difference in the responsiveness of the four PGAs. Bimatoprost had a better IOP-reducing efficacy than latanoprost. There was no significant difference in the IOP-reducing efficacy of travoprost, latanoprost, and tafluprost. In LNRs, the responsiveness of bimatoprost, travoprost, and latanoprost did not show statistical differences. Bimatoprost reduced IOP with a greater extent than latanoprost and travoprost in LNRs, while there was no significant difference in the IOP-reducing efficacy of travoprost and latanoprost. No serious adverse events occurred with the treatment of the four PGAs. The prevalence of conjunctival hyperemia due to bimatoprost or tafluprost was significantly higher than that of latanoprost. Other adverse events had no significant difference between the four drugs.

**Conclusion:**

The existing studies cannot prove that latanoprost, travoprost, bimatoprost, and tafluprost have different responsiveness in OAG/OHT patients. Switching to bimatoprost or travoprost cannot achieve a significant improvement in responsiveness in LNRs. Bimatoprost has a better IOP-reducing efficacy than latanoprost and travoprost. No serious adverse events occurred during treatment with any medication we studied.

## 1. Introduction

Glaucoma is the most common irreversible blindness-inducing disease on a global scale, characterized by a chronic and progressive optic neuropathy and visual field loss [1]. Intraocular pressure (IOP) is the main risk factor for optic nerve damage, and regulation of IOP is the only clinically proven treatment that can delay disease progression [2]. Previous research has demonstrated that lowering of the IOP by 1 mmHg can reduce the risk of glaucomatous progression by approximately 10% [3]. Topical use of ocular hypotensive agents is typically the first therapeutic option in glaucoma. Since the 1990s, prostaglandin F2*α* analogues (PGAs) have gradually replaced *β*-blockers as the first-choice therapy due to their high clinical efficacy to reduce IOP, minimal side effects, and once-daily dosage regimens, consequently facilitating patient compliance [4, 5].

PGAs mainly decrease IOP by increasing outflow facility through an IOP-independent uveoscleral pathway. Some studies have revealed that they can also affect the IOP-dependent conventional trabecular meshwork (TM) outflow pathway [5, 6]. Among the PGAs used in the clinic, latanoprost and travoprost are ester prodrug analogs of prostaglandin F2*α* (PGF2*α*). Though usually classified as prostaglandin analogues, bimatoprost is an amide prodrug of 17-phenyl-PGF2*α* (similar to PGF2*α*) [1, 7]. Tafluprost is a unique PGF2*α* analog. The major modification of tafluprost is the substitution of the C-15 hydrogen and hydroxyl group with two fluorine atoms [8]. PGAs can bind to prostaglandin receptors EP and FP in the ciliary muscle, induce ciliary muscle relaxation, and increase uveoscleral outflow facility. These drugs also increase cell contractility of the TM as well as decrease endothelial cell contractility within Schlemm's canal, mediating aqueous humor outflow through the conventional pathway [9]. PGAs can degrade the extracellular matrix (ECM), which results in ECM turnover in the uvea and TM and ultimately reduces outflow resistance [10].

PGAs have been proven effective in decreasing IOP and are widely used in the treatment of glaucoma. However, after PGA therapies, some patients do not demonstrate a significant reduction in IOP,or fail to achieve the target IOP. Such patients are usually defined as nonresponders. Although there is no clear definition, nonresponders typically refer to patients with an IOP reduction of <15% from baseline after treatment [11]. It was reported that, in American and European populations, 12–41% of patients with glaucoma demonstrated low response to latanoprost, and such patients are defined as latanoprost nonresponders (LNRs). In Singapore, these data are approximately 5.4% [12]. Martínez and colleagues [13] reported that approximately 11% of Hispanics are LNRs. It was reported that the efficacy of latanoprost is significantly undermined in elderly patients and in European and American populations [4]. However, other studies revealed that age and baseline IOP are not factors affecting patient responsiveness to PGAs [14, 15]. Several studies demonstrated that replacing latanoprost with bimatoprost or travoprost can further decrease IOP in LNRs [16–18]. This can be due to varying PGA not acting on the same receptor [19]. Some researchers postulated that bimatoprost interacts with a unique receptor that is unassociated with other PGA receptors. However, this “undefined receptor” has not yet been cloned [1, 10].

This meta-analysis aimed to evaluate the responsiveness of latanoprost (0.005 mg/mL), travoprost (0.004 mg/mL), bimatoprost (0.03 mg/mL), and tafluprost (0.0015 mg/mL) eye-drop-based therapies in patients with open-angle glaucoma (OAG) or ocular hypertension (OHT) and whether substituting latanoprost with other PGAs can further reduce the IOP in LNRs. This study also evaluated the IOP-reducing efficacy and safety of the four PGAs in patients with OAG or OHT.

## 2. Materials and Methods

### 2.1. Literature Search Strategy

A thorough literature search was carried out separately by two researchers on PubMed, Embase, and the Cochrane Controlled Trials Register. The publication time of the selected articles was before 1990, with the type of study restricted to randomized controlled trials (RCTs). The keyword input strategy was “open angle glaucoma” OR “ocular hypertension” AND “latanoprost” OR “travoprost” OR “bimatoprost” OR “tafluprost.” Manual examination of reference lists was carried out, for relevant original research to supplement the study. Following reading the title and abstract, whenever a research study article was deemed relevant to this research, the full text was read.

### 2.2. Inclusion Criteria

- Research type: RCT
- Research object: patients with mild to moderate OAG/OHT and IOP between 21 and 39 mmHg after drug washing
- Research content: comparing the responsiveness of latanoprost, travoprost, bimatoprost, and tafluprost in OAG/OHT patients or LNRs
- The subjects did not undergo any eye surgery within 1 year before treatment
- Follow-up time ≥1 month

### 2.3. Exclusion Criteria

- Nonclinical research, animal research, retrospective research, case report, and review
- Less than 10 patients in each study group
- Repeated data
- Severe OAG with uncontrollable IOP, angle-closure glaucoma, neovascular glaucoma, uveitic glaucoma, and patients who have undergone any eye surgery within one year before participating in the research
- Follow-up time <1 month
- Drugs other than the four drugs studied in this meta-analysis were added to the research plan or combined with other antihypertensive treatments

### 2.4. Data Extraction and Clinical Endpoints

Two researchers extracted metadata separately. Any disagreements were submitted to the third senior researcher for adjudication. The data extracted from the trials included the first author, publication year, blind status, research design (parallel group or crossover study), treatment, single center or multicenter, sample size, type of glaucoma, age, sex, ethnicity, duration of follow-up, number of patients lost to follow-up, and LNR status.

The primary clinical endpoint was the number of responders at the end of each study. A responder was defined as a patient with an IOP reduction of ≥15% or ≥20% from baseline, after therapy. When both the data of IOP reduction ≥15% or 20% were described in a study, the data of IOP reduction ≥20% were extracted. The secondary clinical endpoint was the IOP reduction at the endpoint from baseline. Safety evaluation included five common adverse events: conjunctival hyperemia, hypertrichosis, ocular burning, ocular itching, and foreign-body sensation.

### 2.5. Quality Assessment

Two researchers blindly evaluated the quality of the studies reported in the scientific literature with the Methodological Quality Assessment (Modified Jadad Score). If the results differed, it was resolved through discussion with a third senior researcher. The quality assessment included four items: random sequence generation (appropriate: 2, unclear: 1, and inappropriate: 0), concealment of allocation (appropriate: 2, unclear: 1, and inappropriate: 0), blind (double blind: 2, single blind: 1, and open label: 0), and withdrawals and dropouts (described: 1 and not described: 0). If the Jadad score of a document was ≤3, we defined the study as “low quality.” If the score was between 4 and 7, we defined the research as “high quality.”

### 2.6. Data Analysis

The Stata SE-64® software (StataCorp, College Station, TX, USA) was used for data analysis. We used the relative risk (RR) to summarize dichotomous results and the weighted mean difference (WMD) to summarize continuous results. When the IOP reduction and the standard deviation (SD) value were not available from the original document, we utilized the following formula to calculate [20]:

(1) $$\begin{array}{c} \Delta \text{IOP}={\text{IOP}}_{\text{baseline}}-{\text{IOP}}_{\text{end point}}\\ {\text{SD}}_{\Delta \text{IOP}}=\sqrt{\left(\text{SD baseline}2+\text{SD end point}2-\text{SD baseline}\times \text{SD end point }\right)}. \end{array}$$

The heterogeneity was analyzed by I^2^ statistics. *P* < 0.05 was defined as an index of heterogeneity. When I^2^ > 50%, we considered that heterogeneity was confirmed and we chose a random-effects model for pooling the data in such a situation. When I^2^ ≤ 50%, we chose a fixed-effect model. Due to the limitation of the number of included studies, we did not perform sensitivity analysis and publication bias evaluation.

## 3. Results

### 3.1. Study Selection

**Figure 1:**
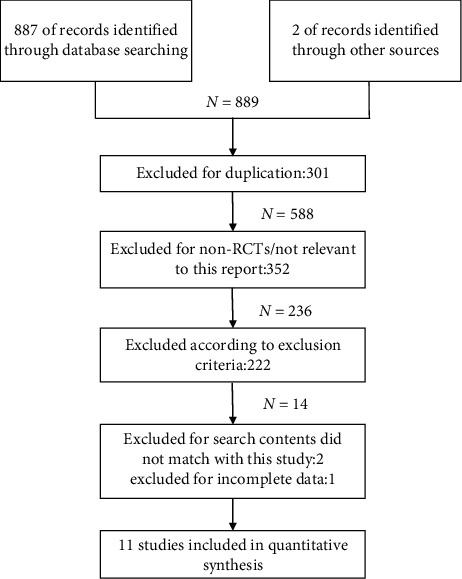
Flow chart of the literature search process.

A total of 889 documents were retrieved and 588 documents remained (following removal of duplicates), of which 236 documents were initially selected following reading the title and abstract. According to the applied inclusion and exclusion criteria, we selected 14 documents deemed as relevant and censored the full text, including references. Two articles were excluded as the research content did not match with this study. One article was excluded due to incomplete data. Finally, 11 articles containing 10 RCTs were included in this meta-analysis [8, 11, 16–18, 21–26]. The detailed search strategy is outlined in Figure 1. Choplin [11] and Noecker [21] studied the same population, though the authors extracted differing outcome variables. A total of 1381 patients were included, with an average age of 60.1 years, of which 45.1% were men. The follow-up time range was from one to six months. Race included white, black, Hispanic, Asian, and Native Hawaiian. The included studies did not analyze patient compliance issues. However, since all the included studies were prospective RCTs and the patient data lost to follow-up were excluded, the analytical assumption placed in this study was that there existed full patient compliance. The detailed data of each study are highlighted in Table 1.

### 3.2. Quality Assessment

According to the revised Jadad scale, two of the studies were defined as “low quality” and the other eight studies were defined as “high quality.” The average score of the included studies was five points. Due to the limitation of the number of included studies, we did not employ a funnel plot to assess publication bias. The specific score of each study is highlighted in Table 2.

### 3.3. Responsiveness and Efficacy of PGAs in OAG/OHT

#### 3.3.1. Responsiveness of PGAs in OAG/OHT

Two trials compared the responsiveness of bimatoprost and latanoprost in OAG/OHT. One trial compared bimatoprost and travoprost in OAG/OHT. Heterogeneity test results across the bimatoprost group and latanoprost group were chi square = 19.23, *P* ≤ 0.001, and I^2^ = 94.8%. We used the randomized effect model for analysis. Three trials compared the responsiveness of tafluprost and latanoprost, and one trial compared tafluprost and travoprost in OAG/OHT. Heterogeneity test results across the tafluprost and latanoprost study groups were chi square = 2.32, *P*=0.314, and I^2^ = 13.7%. The fixed-effect model was employed for analysis. The results were expressed by RR and 95% confidence interval. The responsiveness of bimatoprost was higher than that of latanoprost and travoprost, though the difference was not statistically significant (bimatoprost vs. latanoprost: RR = 1.301, 95% CI: 0.711–2.380, *P*=0.394; bimatoprost vs. travoprost: RR = 1.208, 95% CI: 0.964–1.514, *P*=0.101). The responsiveness of tafluprost was higher than that of latanoprost and travoprost, while the variation was also not statistically significant (tafluprost vs. latanoprost: RR = 1.101, 95% CI: 0.973–1.245, *P*=0.127; tafluprost vs. travoprost: RR = 1.081, 95% CI: 0.771–1.516, *P*=0.652). Detailed data are listed in Table 3.

#### 3.3.2. Efficacy of PGAs in OAG/OHT

One trial compared the IOP-decreasing effect of bimatoprost and latanoprost in OAG/OHT. Three trials compared the IOP-reducing effect of tafluprost and latanoprost; one trial compared the IOP-reducing effect of tafluprost and travoprost in OAG/OHT. Reduction of IOP from baseline is listed in Table 4. Heterogeneity test results across tafluprost and latanoprost study groups were chi square = 0.19, *P*=0.909, and I^2^ = 0.0%. The fixed-effect model was employed for analysis. The results were expressed by WMD and 95% confidence interval. Efficacy outcome revealed a statistically significant difference between bimatoprost and latanoprost (bimatoprost vs. latanoprost: WMD = 1.000, 95% CI: 0.130–1.870, *P*=0.024), suggesting that bimatoprost was more effective than latanoprost in lowering IOP. There was no significant difference in the IOP-reducing efficacy of travoprost, latanoprost, and tafluprost (tafluprost vs. travoprost: WMD = 0.100, 95%CI: −1.414–1.614, *P*=0.897; tafluprost vs. latanoprost: WMD = 0.534, 95%CI: −0.168–1.236, *P*=0.136). Detailed data are listed in Table 5. Due to lack of original data, we did not compare the efficacy of bimatoprost and travoprost, the efficacy of travoprost and latanoprost, or the efficacy of tafluprost and bimatoprost in OAG/OHT.

### 3.4. Responsiveness and Efficacy of PGAs in LNRs

#### 3.4.1. Responsiveness of PGAs in LNRs

Two trials compared the responsiveness of bimatoprost and latanoprost in LNRs (heterogeneity test: chi square = 6.54, *P*=0.011, I^2^ = 84.7%). Two trials compared the responsiveness of bimatoprost and travoprost in LNRs (heterogeneity test: chi square = 2.49, *P*=0.115, I^2^ = 59.8%). These two analyses employed the randomized effect model. One trial compared the responsiveness of travoprost and latanoprost in LNRs. Responsiveness outcome showed no significant difference between the three PGAs (bimatoprost vs. latanoprost: RR = 4.934, 95% CI:0.139–175.638, *P*=0.381; bimatoprost vs. travoprost: RR = 1.361, 95% CI:0.703–2.635, *P*=0.360; travoprost vs. latanoprost:RR = 1.401, 95% CI:0.685–2.864, *P*=0.355). Detailed data are listed in Table 6. Due to lack of original data, the responsiveness of tafluprost could not be evaluated in LNRs.

#### 3.4.2. Efficacy of PGAs in LNRs

Two trials compared the efficacy of bimatoprost and latanoprost in LNRs (heterogeneity test: chi square = 9.63, *P*=0.002, I^2^ = 89.6%). This analysis employed the randomized effect model. Two trials compared the efficacy of bimatoprost and travoprost in LNRs (heterogeneity test: chi square = 0.01, *P*=0.944, I^2^ = 0.0%). In this case, the fixed-effect model was utilized. One trial compared the efficacy of travoprost and latanoprost in LNRs. Reduction of IOP from baseline is listed in Table 7. Efficacy outcome revealed that bimatoprost provided a greater reduction in IOP than latanoprost and travoprost, though there was no statistically significant difference in drug efficacy between travoprost and latanoprost (bimatoprost vs. latanoprost: WMD = 4.153, 95% CI: 0.245–8.062, *P*=0.037; bimatoprost vs. travoprost: WMD = 0.695, 95%CI:0.114–1.277, *P*=0.019; travoprost vs. latanoprost: WMD = 1.400, 95%CI:−1.360–4.160, *P*=0.320). Detailed data are listed in Table 8.

### 3.5. Safety

No serious adverse reactions occurred on patient treatment with the four PGAs. The most common adverse event was conjunctival hyperemia. The prevalence of conjunctival hyperemia was significantly higher in bimatoprost and tafluprost than latanoprost (bimatoprost vs. latanoprost: RR = 2.556, 95% CI: 1.844–3.542, *P* ≤ 0.001; tafluprost vs. latanoprost: RR = 1.779, 95% CI: 1.057–2.995, *P*=0.030). The prevalence of conjunctival hyperemia was also higher in bimatoprost than travoprost though the difference was not statistically significant (bimatoprost vs. travoprost: RR = 1.471, 95% CI: 0.676–3.200, *P*=0.330). There was no statistically significant difference in the prevalence of burning-eye sensation between bimatoprost and latanoprost and tafluprost and latanoprost (bimatoprost vs. latanoprost: RR = 1.169, 95%CI: 0.436–3.132, *P*=0.757; tafluprost vs. latanoprost: RR = 0.990, 95%CI: 0.151–6.477, *P*=0.991) and no statistically significant difference in the prevalence of foreign-body sensation between bimatoprost and travoprost and tafluprost and latanoprost (bimatoprost vs. travoprost: RR = 0.325, 95%CI: 0.034–3.080, *P*=0.327; tafluprost vs. latanoprost: RR = 0.497, 95%CI: 0.174–1.425, *P*=0.193). In addition, there was no statistically significant difference in the prevalence of hypertrichosis (bimatoprost vs. latanoprost: RR = 0.562, 95%CI: 0.002–160.502, *P*=0.842; bimatoprost vs. travoprost: RR = 5.152, 95%CI: 0.250–106.293, *P*=0.288) and itching (bimatoprost vs. latanoprost: RR = 0.345, 95%CI: 0.115–1.031, *P*=0.057; bimatoprost vs. travoprost: RR = 1.304, 95%CI: 0.301–5.641, *P*=0.723) in bimatoprost, travoprost, and latanoprost. There was no statistically significant difference in the prevalence of itching between tafluprost and latanoprost (tafluprost vs. latanoprost: RR = 0.985, 95%CI: 0.349–2.775, *P*=0.977). Detailed data are listed in Table 9.

## 4. Discussion

Presently, five categories of antihypertensive drugs are clinically used to treat glaucoma: *β*-blockers, carbonic anhydrase inhibitors (CAIs), sympathomimetics, *α*-2 adrenergic agonists, and PGAs [1, 27]. PGAs have proven to be more effective than the other four drug classes in reducing IOP and are widely used as first-line of treatment for glaucoma [4, 27, 28]. However, previous studies did not find significant differences in the efficacy of PGAs [16, 23, 27].

PGAs currently available on the market include latanoprost, travoprost, bimatoprost, tafluprost, and unoprostone. Latanoprost and travoprost are synthetic ester prodrugs of natural PGF2*α*. Their hydrolysis products manifest their action through binding to the FP and EP receptors in the ciliary body, causing relaxation of the ciliary muscle and increasing outflow facility of the IOP-independent uveoscleral pathway. Such by-products can also increase TM pathway outflow facility, albeit mainly through the uveoscleral pathway [18, 29–31]. With a unique chemical structure different from other PGAs, bimatoprost is a synthetic anamide prodrug of 17-phenyl-PGF2*α* [5]. Bimatoprost can act as a complete molecule to decrease IOP, without hydrolysis, to be biologically active as latanoprost and travoprost [17, 18]. The hydrolysate of bimatoprost (17-phenyl PGF2*α*, also named bimatoprost acid) is also an effective FP receptor agonist that can promote outflow facility by activating the FP receptor [7]. Several studies demonstrated that bimatoprost can reduce IOP through a pathway that is independent of FP receptors [32]. The major modification of tafluprost is the substitution of the C-15 hydrogen and hydroxyl group with two fluorine atoms [8]. Its metabolite, tafluric acid, is a FP receptor agonist with an affinity for the FP receptor that is 12-fold higher than the affinity of the latanoprost metabolite [33]. Unoprostone is also a PGF2*α* analogue. However, due to the docosanoid structure, it has very low affinity for the prostaglandin receptor [34]. Early animal studies suggested that unoprostone can increase uveoscleral pathway outflow by activating the FP receptor [35]. Recent studies found that unoprostone increases TM path outflow through stimulating calcium-ion- (Ca2+-) activated BK- and CIC-2-type channels [34, 36]. Currently, the Food and Drug Administration (FDA) has removed the description of this drug as a PGA [36]. Due to the limitations of existing studies, we did not analyze the responsiveness of unoprostone.

It has been reported that 15% of patients do not adequately respond to PGAs, which could be partly due to single nucleotide polymorphisms (SNPs) in genes encoding matrix metalloproteinases (MMPs) and PGF2*α* receptor (PTGFR) [5]. Sakurai et al. [37] reported the SNPs rs3753380 and rs3766355 in the promoter and intron 1 region of the PTGFR gene in healthy Japanese volunteers can cause downregulation of receptor expression after a short duration of latanoprost treatment, undermining the efficacy. They also found an association between the SNP rs12093097 in the PTGFR gene and the response to latanoprost in patients with glaucoma or OHT [38]. Ussa et al. [14] demonstrated that the SNPs rs6686438 and rs10786455 in PTGFR are likely to be related to a positive response to latanoprost, and SNPs rs3753380, rs6672484, and rs11578155 are likely to be related to a negative response to latanoprost in patients with OAG. This study revealed that SNPs in the gene encoding MMP-1 also influence the response to latanoprost. MMPs, which are neutral proteases expressed by TM and uveoscleral tissues, can initiate degradation of ECM and regulate outflow resistance [10]. SNPs also affect the responsiveness to other ocular hypotensive agents in patients with glaucoma. Cytochrome P450 (CYP) 2D6 Arg296Cys is a polymorphic site on the CYP2D6 gene [39]. It has been reported that the Arg296Cys travoprost genotype can increase CYP2D6 activity, enhance timolol metabolism, and thus, reduce timolol therapeutic effects [39].

Several studies have found differences in responsiveness and efficacy of multiple PGAs in OAG/OHT [11, 16, 22, 40], with variations in the molecular structure and mechanism of action of PGAs being a potential reason. Latanoprost and travoprost are synthetic ester prodrugs of natural PGF2*α*, which necessitate hydrolysis into the active form during the corneal passage [18, 29–31]. The lack of response to latanoprost may be due, in part, to poor de-esterification of the prodrug to the pharmacologically active free fatty acid [4]. Tafluprost requires to be metabolized by corneal esterase to exert its antihypertensive effect. The difference in corneal permeability could be a factor related to the variation in responsiveness [26]. Bimatoprost, a fatty acidamide, is an amide prodrug of 17-phenyl-PGF2*α* [5]. Fatty-acid amides are neutral lipids without the negative charge associated with the carboxylic acid group of fatty acids such as PGF2*α*, latanoprost, and travoprost. This is an important structural difference of bimatoprost from other PGAs [7]. Bimatoprost can manifest its IOP-lowering ability as a complete prostaglandin-like molecule, without any metabolic conversion [17, 18]. The hydrolysate of bimatoprost (17-phenyl PGF2*α*, also named bimatoprost acid) is also an effective FP receptor agonist and can promote outflow facility [7]. Several studies suggested that bimatoprost has unique pharmacological effects. Besides the known prostanoid receptors, it can also act on a dedicated prostamide-sensitive receptor that can partially explain the unique bimatoprost effects compared to other PGAs [5, 7, 10]. Chen et al. [32] reported that bimatoprost can exert its effects on the human *T* lymphoblast (peripheral blood acute lymphoblastic leukemia, MOlatanoprost-3) cells, while qPCR analysis had proven that MOlatanoprost-3 cells expressed no FP or thromboxane A2 receptors (TP). This result supported the hypothesis that bimatoprost can bind to a unique receptor beyond FP and TP. However, this unique “undefined receptor” has not been cloned in studies thus far [1].

In glaucoma, the increase of ECM in TM can also induce the obstruction of outflow facility. MMPs can degrade the ECM in the TM and uveal region, subsequently skewing the resistance to aqueous humor outflow [29]. Heo et al. [6] found that latanoprost and bimatoprost can effectively upregulate the activities of MMP-1 and MMP-9 in human TM. Furthermore, in the study of Li et al. [10] on immortalized human TM (iHTM) cells, it was found that latanoprost and bimatoprost significantly upregulated the expression of transcription factors c-fos and MMP-9 and downregulated the expression of the tissue inhibitor of metalloproteinase 4 (TIMP-4) simultaneously, consequently promoting the degradation of ECM in the TM and increased outflow facility. This study also revealed that fibronectin mRNA expression was upregulated by latanoprost though downregulated by bimatoprost. Fibronectin can increase the stiffness of the TM, change the resistance to aqueous humor outflow, and finally, increase IOP [41]. Several studies found the fibronectin levels in aqueous humor of glaucomatous patients were nearly seven-fold higher than those of patients with cataracts [42]. The degradation of fibronectin will induce ECM turnover in the TM and inner wall of Schlemm's canal, ultimately affecting the resistance of outflow facility [43]. This can constitute the reason why bimatoprost has enhanced efficacy properties than latanoprost: although both PGAs can activate ECM degeneration, the increased expression of fibronectin might impair the IOP-reducing efficacy of latanoprost. In addition, bimatoprost can significantly downregulate the expression of aquaporin-1 (AQP1) in iHTM [10]. AQPs are 10 AQP families involved in water transportation, which are expressed in multiple organizations [44]. In ocular tissues, AQP1 is expressed in the lens epithelial cells (LECs), ciliary epithelium, and iris. In LECs, the high expression of AQP1 will increase the penetration of the aqueous humor into the lens, resulting in thickening of the lens and, consequently, obstructing the outflow of the aqueous humor [45]. In the iris and ciliary epithelium, AQP1 plays a role in the production of the aqueous humor by transporting water out of the ciliary epithelium [46]. Compared with normal individuals, the IOP of AQP1-knockout mice was significantly decreased [47]. Downregulated AQP1 expression could be one of the reasons why bimatoprost has a higher IOP-reducing efficacy than latanoprost. However, AQP1 is also expressed in the TM and can regulate the volume of trabecular cells [48] and mediate cytoskeleton remodeling [49] and cell migration/proliferation [49, 50] through interactions with *β*-catenin. Zhao et al. [46] demonstrated that AQP1 expression was downregulated in TM cells exposed to Endothelin-1 (ET-1), causing glaucomatous changes such as actin fiber reorganization, collagen production, extracellular matrix deposition, and contractility alteration of TM cells, eventually leading to an increase in IOP. The ET-1-induced actin fiber reorganization in human TM cells can be significantly reversed through transfection with an adenoviral vector encoding for full-length AQP1 [46]. Therefore, whether decreasing the expression of AQP-1 can help reduce IOP remains to be explored.

The results of this meta-analysis reveal that bimatoprost responsiveness in patients with OAG/OHT is higher than for latanoprost and travoprost, although this advantage is not statistically significant. Tafluprost responsiveness in patients with OAG/OHT is higher than for latanoprost and travoprost, though this is not statistically significant either. Due to the lack of original data, we did not directly compare the responsiveness of latanoprost and travoprost and bimatoprost and tafluprost in OAG/OHT. Bimatoprost has a higher efficacy for reducing IOP than latanoprost. There is no significant variation in the IOP-reducing efficacy of travoprost, latanoprost, and tafluprost. Due to the lack of original data, we did not compare the IOP-reducing efficacy of bimatoprost and travoprost and travoprost and latanoprost, as well as bimatoprost and tafluprost in OAG/OHT. In OAG/OHT patients who do not respond to latanoprost, switching to bimatoprost or travoprost can increase the percentage of patients demonstrating response, with the response to bimatoprost higher than that of travoprost. However, this improvement does not possess statistical significance either. Compared with latanoprost and travoprost, bimatoprost can more effectively lower IOP in LNRs, though there is no significant difference in travoprost and latanoprost efficacy. All four PGA classes exhibited good safety profiles, with no serious, vision-impairing adverse events taking place. The most common adverse event was conjunctival hyperemia. The prevalence of conjunctival hyperemia in patients using bimatoprost or tafluprost is significantly higher than that using latanoprost. The prevalence of conjunctival hyperemia in patients using bimatoprost is also higher than that using travoprost, albeit with no statistical difference. The other adverse events (burning, foreign-body sensation, eyelash hypertrichosis, and itching) demonstrated nonsignificant differences in the three classes of PGAs. The studies conducted by Konstas [22] and Kammer [16] also confirmed that there was no significant difference in the incidence of skin pigmentation among patients using bimatoprost, latanoprost, and travoprost, although the data were insufficient and we did not conduct a meta-analysis.

In the two studies studying the responsiveness of bimatoprost in LNRs, the crossover study conducted by Gandolfi et al. [18], revealed that, among 15 patients who did not respond to latanoprost after 6–8 weeks of treatment, none of the patients exhibited response on continuing latanoprost treatment for one month, while 13 patients exhibited response after converting to bimatoprost treatment for one month. However, Blondeau et al. [17] found that, after one-month treatment of bimatoprost, 13 out of 31 LNRs exhibited response to bimatoprost, with 9 out of 29 converting into responders after continuing latanoprost treatment for one month. The study authors believed that this increase in responsiveness could be partly due to median regression and the Hawthorne effect. The Hawthorne effect is that when subjects know they participate in a study, their behavior may change and demonstrate enhanced patient compliance [17, 51]. This means that a subgroup of patients might not actually lack in response. Their poor compliance during nonstudy time periods affects the efficacy of the drug. The bimatoprost implant Durysta™, developed by Allergan (USA), can avoid the impact of patient compliance on efficacy. It can be implanted into the anterior chamber and release bimatoprost slowly and continuously, effectively reducing IOP for 4–6 months after implantation [52]. This therapeutic measure could effectively enhance the patient's response to such drugs. In 2020, bimatoprost implants have been approved in the USA for OAG/OHT treatment [53].

Preservative use is an important factor affecting adverse reactions. All of the four PGA eyedrops studied in this meta-analysis contain preservatives. Benzalkonium chloride (BAK) is a common preservative and is widely used as a component of eyedrops for its antibacterial properties [54]. However, through cellular apoptosis and neurotoxicity, BAK can possibly induce ocular surface diseases (OSDs), leading to several symptoms including dryness, irritation, burning sensations, foreign-body sensations, photophobia, and eye fatigue [55, 56]. These adverse reactions reduce patient tolerability, leading to decreased patient compliance, and ultimately influence the effectiveness of topical glaucoma therapy [55]. A range of low-toxicity preservatives have been developed to replace BAK, such as SofZia and Polyquaternium-1 (also named polyquad) [57]. Kumar [57] reported that Ocular Surface Disease Index (OSDI) scores were significantly lower in polyquad-preserved travoprost in comparison to BAK-preserved travoprost, with comparable IOP-reducing efficacy. Aihara [54] discovered that switching from BAK-preserved latanoprost to SofZia-preserved travoprost can ameliorate chronic superficial punctate keratitis and there was no significant change in conjunctival hyperemia, tear breakup time (TBUT), or IOP. Despite having low toxicity, such novel preservatives can still cause adverse reactions [58, 59]. Preservative-free eyedrops may bring enhanced safety. Preservative-free tafluprost (PF-tafluprost) is the first preservative-free formulation for a PGA preparation [56, 60]. Hamacher [60] demonstrated that PF-tafluprost had an equivalent IOP-reducing efficacy compared to preservative-containing tafluprost (PC-tafluprost) and is well tolerated. Ruangvaravate [56] demonstrated that switching from other preservative-containing prostaglandins to PC-tafluprost and PF-tafluprost both increased TBUT in glaucoma patients with OSD, while PF-tafluprost had better tear quality versus PC-tafluprost. Therefore, PF-tafluprost should be especially beneficial for patients with preexisting OSD. Several studies found that PF-latanoprost had an equivalent IOP-reducing efficacy compared to PC-latanoprost, although it was better tolerated [55, 61]. Preservative-free eyedrops could possibly be the future development tendency for antiglaucoma drugs.

This meta-analysis has several certain limitations. Firstly, the number of included studies is small, which could lead to biased results. Furthermore, this study lacks a direct comparison of the responsiveness and safety of travoprost and latanoprost and bimatoprost and tafluprost, in patients with OAG/OHT. The population demographics included in this meta-analysis included white, black, Hispanic, and Asian. However, we did not conduct subgroup analysis for race due to the limitation of original data. The length of follow-up time of the included studies ranged from one to six months, for it is highly challenging to study the long-term efficacy and responsiveness of the four PGAs in OAG/OHT populations. Finally, there are five PGAs used in clinical treatments, though, due to the lack of original literature, we did not analyze the responsiveness and efficacy of unoprostone.

## 5. Conclusions

In essence, existing studies highlight that latanoprost, travoprost, bimatoprost, and tafluprost do not have statistically significant differences in responsiveness within the OAG/OHT patient population. OAG/OHT patients who are LNRs cannot get a significant increase in response by switching to travoprost or bimatoprost. The IOP-reducing efficacy of bimatoprost is significantly higher than that of latanoprost. There is no significant difference in the IOP-reducing efficacy of travoprost, latanoprost, and tafluprost. All the four PGAs have good safety. The prevalence of conjunctival hyperemia due to bimatoprost or tafluprost is higher than that of latanoprost. Other adverse events show no significant difference between the four drugs.

We hope that additional high-quality and large-sample RCTs can be carried out in the near future to compare the responsiveness and IOP-reducing efficacy of different PGAs in the OAG/OHT patient population.

## Figures and Tables

**Table 1:** Characteristics of studies included in the meta-analysis.

Author	Year	Blind	Design	Center	Treatment	Number of patients	Age (years)	Type of diagnosis	LNR	Male/female	Race	Follow-up (months)	Lost to follow-up
Blondeau et al. [17]	2019	No	PG	Single	LAT vs. TRA vs. BIM	83	68.8	OAG and OHT	Yes	43/40	White	1	0
Choplin et al. [11]	2004	Single	PG	Multi	BIM vs. LAT	269	61.3	OAG and OHT	No	103/169	White (83%), black (11%), and Hispanic (6%)	6	20/269 (7.43%)
Noecker et al. [21]	2003	Single	PG	Multi	BIM vs. LAT	269	61.3	OAG and OHT	No	103/169	White (83%), black (11%), and Hispanic (6%)	6	20/269 (7.43%)
Kammer et al. [16]	2010	Single	PG	Multi	BIM vs. TRA	266	63.0	OAG and OHT	Yes	119/147	White (57.3%), black (27.5%), Hispanic (12.2%), asian (1.5%), and native Hawaiian (1.5%)	3	7/266 (2.63%)
Konstas et al. [22]	2007	Single	CR	Multi	BIM vs. LAT	129	66.5	XFG	No	N. A	White	3 × 2	6/129 (4.66%)
Noecker et al. [23]	2006	Single	PG	Multi	BIMvs. TRA	94	63.4	OAG and OHT	No	37/57	Black	3	3/94 (3.20%)
Gandolfi and Cimino [18]	2003	Double	CR	Single	BIM vs. LAT	15	62.0	OAG and OHT	Yes	6/9	N. A	1 × 2	0
Kuwayama and Komemushi [24]	2008	Single	PG	Multi	TAF vs. LAT	125	59.0	OAG and OHT	No	55/42	Asian	1	20/125 (16.0%)
Traverso et al. [25]	2010	Double	PG	Multi	TAF vs. LAT	38	N. A	OAG	No	12/26	White	1.5	2 (5.26%)
Ge et al. [8]	2015	Single	PG	Multi	TAF vs. LAT	246	44.0	OAG and OHT	No	125/71	Asian	1	21/246 (8.53%)
Mizoguch et al. [26]	2012	No	CR	Multi	TAF vs. TRA	116	69.4	NTG	No	23/67	Asian	3 × 2	20/116 (17.2%)

^*∗*^PG: parallel group, CR: crossover, XFG: exfoliative glaucoma, BIM: bimatoprost, TRA: travoprost, LAT: latanoprost, TAF: tafluprost.

**Table 2:** Quality assessment of the included studies.

		Random sequence generation	Concealment of allocation	Double blind	Withdrawals and dropouts	Total score
Blondeau et al. [17]	2019	2	0	0	1	3
Noecker et al. [21]	2003	2	2	1	1	6
Kammer et al. [16]	2010	2	2	1	1	6
Konstas et al. [22]	2007	1	1	1	1	4
Noecker et al. [23]	2006	2	1	1	1	5
Gandolfi and Cimino [18]	2003	1	1	1	1	4
Kuwayama and Komemushi [24]	2008	2	2	1	1	6
Traverso et al. [25]	2010	2	2	2	1	7
Ge et al. [8]	2015	2	2	1	1	6
Mizoguch et al. [26]	2012	1	1	0	1	3

**Table 3:** Responsiveness of PGAs in OAG/OHT.

	Number of trials	BIM (N)	TRA (N)	LAT (N)	TAF (N)	RR (95%CI)	Test for heterogeneity	Test for overall effect
BIM vs. LAT	2	257	0	261	0	1.301 (0.711, 2.380)	*Q* = 19.23, *P* ≤ 0.001	*Z* = 0.85, *P*=0.394
BIM vs. TRA	1	47	44	0	0	1.208 (0.964, 1.514)	N. A	*Z* = 1.64, *P*=0.101
TAF vs. LAT	3	0	0	193	186	1.101 (0.973, 1.245)	*Q* = 2.32, *P*=0.314	*Z* = 1.52, *P*=0.127
TAF vs. TRA	1	0	90	0	90	1.081 (0.771, 1.516)	N. A	*Z* = 0.45, *P*=0.652

^*∗*^BIM: bimatoprost, TRA: travoprost, LAT: latanoprost, TAF: tafluprost.

**Table 4:** Reduction of IOP from baseline of PGAs in OAG/OHT.

Author	Year	BIM (n)	TRA (n)	LAT (n)	TAF (n)
Konstas et al. [22]	2007	9.3 ± 3.4 (124)	N. A	8.3 ± 3.6 (125)	N. A
Mizoguch et al. [26]	2012	N. A	2.2 ± 2.2 (90)	N. A	2.3 ± 2.3 (90)
Kuwayama and Komemushi [24]	2008	N. A	N. A	6.2 ± 2.5 (51)	6.6 ± 2.5 (46)
Traverso et al. [25]	2010	N. A	N. A	8.6 ± 3.0 (18)	9.5 ± 3.1 (18)
Ge et al. [8]	2015	N. A	N. A	9.2 ± 4.1 (105)	9.8 ± 4.0 (91)

^*∗*^BIM: bimatoprost, TRA: travoprost, LAT: latanoprost, TAF: tafluprost.

**Table 5:** Efficacy of PGAs in OAG/OHT

	Number of trails	BIM (N)	TRA (N)	LAT (N)	TAF (N)	WMD (95%CI)	Test for heterogeneity	Test for overall effect
BIM vs. LAT	1	124	0	125	0	1.000 (0.130, 1.870)	N. A	*Z* = 2.25, *P*=0.024
TAF vs. TRA	1	90	90	0	0	0.100 (−1.414, 1.614)	N. A	*Z* = 0.13, *P*=0.897
TAF vs. LAT	3	0	0	174	155	0.534 (−0.168, 1.236)	*Q* = 0.19, *P*=0.909	*Z* = 1.49, *P*=0.136

^*∗*^BIM: bimatoprost, TRA: travoprost, LAT: latanoprost, TAF: tafluprost.

**Table 6:** Responsiveness of PGAs in LNRs.

	Number of trials	BIM (N)	TRA (N)	LAT (N)	RR (95%CI)	Test for heterogeneity	Test for overall effect
BIM vs. LAT	2	46	0	44	4.934 (0.139, 175.638)	*Q* = 6.54, *P*=0.011	*Z* = 0.88, *P*=0.381
BIM vs. TRA	2	162	158	0	1.361 (0.703, 2.635)	*Q* = 2.49, *P*=0.115	*Z* = 0.91, *P*=0.360
TRA vs. LAT	1	0	23	29	1.401 (0.685, 2.864)	N. A	*Z* = 0.92, *P*=0.355

^*∗*^BIM: bimatoprost, TRA: travoprost, LAT: latanoprost.

**Table 7:** Reduction of IOP from baseline of PGAs in LNRs.

Author	Year	Bimatoprost (n)	Travoprost (n)	Latanoprost (n)
Gandolfi and Cimino [18]	2003	6.7 ± 1.5 (15)	N. A	0.7 ± 1.0 (15)
Kammer et al. [16]	2010	2.1 ± 2.4 (128)	1.4 ± 2.5 (132)	N. A
Blondeau et al. [17]	2019	4.9 ± 4.6 (31)	4.3 ± 5.3 (23)	2.9 ± 4.7 (29)

**Table 8:** Efficacy of PGAs in LNRs.

	Number of trials	BIM (N)	TRA (N)	LAT (N)	WMD (95%CI)	Test for heterogeneity	Test for overall effect
BIM vs. LAT	2	46	0	44	4.153 (0.245, 8.062)	*Q* = 9.63, *P*=0.002	*Z* = 2.08, *P*=0.037
BIM vs. TRA	2	162	158	0	0.695 (0.114, 1.277)	*Q* = 0.01, *P*=0.944	*Z* = 2.34, *P*=0.019
TRA vs. LAT	1	0	23	29	1.400 (−1.360, 4.160)	N. A	*Z* = 0.99, *P*=0.320

^*∗*^BIM: bimatoprost, TRA: travoprost, LAT: latanoprost.

**Table 9:** Safety analysis for PGAs.

		Number of trials	BIM (N)	TRA (N)	LAT (N)	TAF (N)	RR (95%CI)	Test for heterogeneity	Test for overall effect
Conjunctival hyperemia	BIM vs. LAT	3	272	0	276	0	2.556 (1.844, 3.542)	*Q* = 1.87, *P*=0.392	*Z* = 5.63, *P* ≤ 0.001
	BIM vs. TRA	2	180	180	0	0	1.471 (0.676, 3.200)	*Q* = 0.22, *P*=0.639	*Z* = 0.97, *P*=0.330
	TAF vs. LAT	2	0	0	174	175	1.779 (1.057, 2.995)	*Q* = 0.78, *P*=0.378	*Z* = 2.17, *P*=0.030

Burning	BIM vs. LAT	1	133	0	136	0	1.169 (0.436, 3.132)	N.A.	*Z* = 0.31, *P*=0.757
	TAF vs. LAT	2	0	0	174	175	0.990 (0.151, 6.477)	*Q* = 4.87, *P*=0.027	*Z* = 001, *P*=0.991

Foreign body sensation	BIM vs. TRA	2	180	180	0	0	0.325 (0.034, 3.080)	*Q* = 0.00, *P*=0.961	*Z* = 0.98, *P*=0.327
	TAF vs. LAT	2	0	0	174	175	0.497 (0.174, 1.425)	*Q* = 0.63, *P*=0.429	*Z* = 1.30, *P*=0.193

Hypertrichosis	BIM vs. LAT	2	257	0	261	0	0.562 (0.002, 160.502)	*Q* = 12.79, *P* ≤ 0.001	*Z* = 0.20, *P*=0.842
	BIM vs. TRA	1	131	135	0	0	5.152 (0.250, 106.293)	N.A.	*Z* = 1.06, *P*=0.288

Itching	BIM vs. LAT	1	133	0	136	0	0.345 (0.115, 1.031)	N.A.	*Z* = 1.90, *P*=0.057
	BIM vs. TRA	2	180	180	0	0	1.304 (0.301, 5.641)	*Q* = 1.31, *P*=0.252	*Z* = 0.35, *P*=0.723
	TAF vs. LAT	2	0	0	73	74	0.985 (0.349, 2.775)	*Q* = 0.59, *P*=0.443	*Z* = 0.035, *P*=0.977

^*∗*^BIM: bimatoprost, TRA: travoprost, LAT: latanoprost, TAF: tafluprost.

## Data Availability

The data used to support the findings of this study are available from the corresponding author upon request.
